# Grain Mineral Accumulation Changes in Chinese Maize Cultivars Released in Different Decades and the Responses to Nitrogen Fertilizer

**DOI:** 10.3389/fpls.2019.01662

**Published:** 2020-01-14

**Authors:** Song Guo, Yanhua Chen, Xiaochao Chen, Yanling Chen, Lan Yang, Lifeng Wang, Yusheng Qin, Mingshun Li, Fanjun Chen, Guohua Mi, Riliang Gu, Lixing Yuan

**Affiliations:** ^1^ Soil and Fertilizer Research Institute, Sichuan Academy of Agricultural Sciences, Chengdu, China; ^2^ College of Resources and Environmental Sciences, China Agricultural University, Beijing, China; ^3^ College of Resources and Environmental Sciences, Qingdao Agricultural University, Qingdao, China; ^4^ College of Resources and Environmental, Hunan Agricultural University, Changsha, China; ^5^ Institute of Cereal Crops, Henan Academy of Agricultural Sciences, Zhengzhou, China; ^6^ Institute of Crop Sciences, Chinese Academy of Agricultural Sciences, Beijing, China; ^7^ Center for Seed Science and Technology, College of Agronomy and Biotechnology, China Agricultural University, Beijing, China

**Keywords:** grain, mineral concentration, genotype era, nitrogen, maize breeding

## Abstract

Evaluating changes in the accumulation of grain minerals, including nitrogen (N), copper (Cu), iron (Fe), potassium (K), magnesium (Mg), manganese (Mn), phosphorus (P), and zinc (Zn), across different genotypes can provide valuable information for the development of nutrient-enriched maize varieties. Meanwhile, N rates can affect maize yield and quality, but their effects on element accumulation remain to be elucidated. Here, field experiments were conducted at two locations in China over 2 years (2010 and 2011). Under a normal N application rate (240 kg N ha^−1^), 24 maize cultivars that had been bred and released between 1930 and 2010 were evaluated for the elemental concentrations in the grains. Cultivars Yedan 13 and Zhengdan 958, representing old- and new-era cultivars respectively, were selected to investigate grain element accumulation in response to different levels of N (0, 60, 120, 180, and 240 kg N ha^−1^). The results showed that element concentrations were significantly affected by year, genotype (G), N rates, and N × G interaction. Grain yield tended to increase with the year of cultivar released, while the concentrations of N, Cu, Mn, and Zn in the grain significantly declined in the new-era. The element concentrations of grains were mainly influenced by N rate or N × G interactions. As N levels increased, N, Cu, Fe, Mg, and Mn concentrations rose, while K, P, and Zn concentrations decreased. Compared with old-era cultivars, new-era cultivars showed an increase in grain yield of 25.39%; however, they demonstrated decreases in N, Cu, Fe, K, Mg, P, and Zn concentrations. In the new-era varieties, the reduction in Cu, Fe, K, and P concentrations were significantly exacerbated by high N rates, but this was not the case in the old-era varieties. The concentration of grain Cu, K, Mg, P, and Zn were higher under N-limited condition (N0), but grain yield was also lower. However, the optimal N rate (120–180 kg N ha^−1^) could increase N, Fe, Mg, and Mn concentrations without affecting grain yield in new-era varieties. It is concluded that maize breeding processes have improved grain yield, but reduced grain nutrient element concentrations. Enhanced concentrations of certain elements in maize grain could be achieved with optimal rates of N fertilizer being applied.

## Introduction

Nutrients, such as nitrogen (N), copper (Cu), iron (Fe), potassium (K), magnesium (Mg), manganese (Mn), phosphorus (P), and zinc (Zn), are essential to the growth of higher plants and are crucial to human and animal health (Nuss and Tanumihardjo, 2010). More than three billion people worldwide, mostly in developing nations, suffer poor health and reduced life expectancy due to malnutrition associated with a lack of nutrients (Cakmak et al., 2010). As one of the three major cereal grains, maize (*Zea mays* L.) is essential to food security, animal and human nutrition, and health (DeFries et al., 2015). Accounting for 40% of the world’s cereal crops (Bouis and Welch, 2010), maize provides 15 and 20% of the total proteins and calories, respectively, consumed by the world’s population (Nuss and Tanumihardjo, 2010). Therefore, new ways to improve maize kernel quality are of great importance (Nuss and Tanumihardjo, 2010). Recent approaches to enhance the nutrient accumulation in grain have included genetic improvements, conventional breeding, or agronomy management (Welch and Graham, 2002; Gu et al., 2015).

Positive correlations between nutrient concentrations and one another in wheat and rice grains have been reported (Skrbic and Onjia, 2007; Cakmak et al., 2010; Chatzav et al., 2010; Anandan et al., 2011). For example, significant positive correlations were found between concentrations of P and those of Mg, Fe, Mn, Zn, and K in the grain of a maize recombinant inbred population (Gu et al., 2015). Additionally, the grain Fe and Zn concentrations of 24 maize cultivars were demonstrated to be significantly and positively correlated (P < 0.01; Akinwale and Adewopo, 2016). In contrast, no significant association was found between maize grain Fe and Zn concentrations (Prasanna et al., 2011) and a positive correlation was found between grain P and K concentrations in 12 tropical maize cultivars only during the rainy season (Feil et al., 1990). Studies have revealed that there are extensive genotype variations in crop grain nutrient concentrations (Feil et al., 1990; Banziger and Long, 2000; Oikeh et al., 2003a; Oikeh et al., 2003b; Prasanna et al., 2011). These studies suggest that crops can be bred to be richer in grain nutrient elements, but, in making these genetic improvements, researchers must consider the possibility of interactions (positive or negative) with the environment.

The average yield of commercial maize in the United States has increased markedly, from 1.3 Mg ha^−1^ in 1939 to 9 Mg ha^−1^ in 2012, but to a lesser extent in China, from 1 Mg ha^−1^ in 1949 to 6 Mg ha^−1^ in 2012 (Duvick, 2005; Li and Wang, 2009; Ciampitti and Vyn, 2014). Many researchers have reported a decrease in the N concentration in grain over the years (Duvick, 2005; Chen et al., 2013; Haegele et al., 2013). Higher protein concentrations and lower starch concentrations were found in Chinese hybrids compared with hybrids from the United States released during the 1960s and 2000s (Li et al., 2016). A study involving six maize hybrids released between 1959 and 1988 showed that kernel concentrations of Mg, Cu, and Mn were higher in the older hybrids (Vyn and Tollenaar, 1998). Furthermore, in rice, the concentrations of Zn and Fe in improved cultivars were significantly lower than those in traditional genotypes (Anandan et al., 2011). The exclusive breeding selection for grain yield over quality has led to the decrease in nutrient concentrations in grain over a number of years (Fan et al., 2008; Gu et al., 2015). Research workers reported a low negative correlation between maize grain Fe concentration and yield, but no significant correlation between maize grain Zn concentration and yield (Chakraborti et al., 2009). A negative correlation between some nutrients concentration and yield were also detected in rice (Anandan et al., 2011), whereas significant positive correlations were observed in wheat between grain yield and some nutrients (Fe, Mn, Cu, Zn, and N) concentrations, but not for Mg, K, and P concentrations (Chatzav et al., 2010). A recent report showed the nutrient harvest index values of modern maize hybrids to be N 58%, P 79%, K 33%, Mg 29%, Cu 29%, Fe 18%, Mn 13%, and Zn 62%, respectively (Bender et al., 2013). Therefore, it is necessary to better understand the association between grain nutrient concentrations in maize cultivars and their year of cultivar release.

N is the key limiting nutrient for crop productivity, and N fertilizer application significantly impacts maize yield. In China, N fertilizer input increased by 271% between 1977 and 2005, whereas the total annual grain production increased by only 71% (Yang et al., 2016). When maize yield reaches a certain level, further addition of N fertilizer does not increase yield (Hammad et al., 2011; Chen et al., 2015). Researchers studying the yield and N-use efficiency of maize cultivars released over the past five decades under high/low N applications in China and USA, found that modern maize cultivars had increased their responsiveness to high N and their tolerance to low N, compared with traditional maize cultivars (Chen et al., 2013; Haegele et al., 2013). An increase in yield was exhibited when comparing old-era cultivars (1940–1990) with new-era cultivars (1991–2011) of maize at comparable levels of N uptake, while grain N concentrations were shown to be lower in new-era cultivars than in old-era cultivars (Ciampitti and Vyn, 2012). N levels can influence the grain accumulation and concentration of other nutrients in maize. For example, the Mn, Fe, Zn, and K concentrations were significantly reduced following the addition of N fertilizer, and a significant negative correlation was observed between N fertilizer rates and both Zn and K concentrations in maize grain (Rui et al., 2009). However, some studies have suggested that a continuous input of N fertilizer does not result in reduced grain quality (Losak et al., 2011). Another study showed that the Zn, Fe, Mn, and Cu concentrations in maize grain increased in concert with increased grain yields under optimized N management (Xue et al., 2014). The relationships reported in the literature between N and other nutrients have been found, therefore, to be inconsistent, especially in modern cultivars (Ma and Zheng, 2018). Thus, further investigation is necessary to clarify how increased N rates have influenced the kernel concentrations of other nutrients in maize crops over the decades and how optimal N management improves nutrient concentrations as well as achieving a higher grain yield.

Breeding new genotypes with high grain nutrient concentrations is an effective strategy to solve the problem of nutrient-specific malnutrition, but is a long-term process. Applying N fertilizers is also an effective agronomic strategy for improving grain nutrient concentrations (Kutman et al., 2010). Selecting high-yielding cultivars with high N remobilization efficiency in combination with improved N management can increase both cereal N concentration and grain yield (Chen et al., 2015). In this study, field experiments were conducted at two sites across 2 years (2010 and 2011), using 24 Chinese maize cultivars that were released between 1930 and 2010 and different N rates. Phenotypic data for grain yield and eight nutrient (N, Cu, Fe, K, Mg, Mn, P, and Zn) concentrations were determined. The aims of the study were to evaluate the grain nutrient concentrations in maize cultivars released in different decades and to evaluate the impact of N application rates on nutrient accumulation, while determining the interactions between N and the accumulation of individual elements in the kernels of old- and new-era cultivars. The purpose of the study was also to investigate how cultivar selection and nitrogen management affected maize kernel quality.

## Materials and Methods

### Plant Materials and Experimental Design

Twenty-four cultivars (**Table 1**) that had been released between 1930 and 2010 and grown extensively from 1950 to 2010 in the main maize-growing areas of China (Ci et al., 2012) were planted in Shun-Yi, Beijing (40°07’N, 116°39’E). The physiological maturity date was identified as the date when a black layer was visible at the grain base in 50% of the ears. Plant height was measured from the surface of the ground to the tip of the tassel. N was applied at a rate of 240 kg ha^−1^ at Shun-Yi to study the final nutrient concentrations in the kernels of the different maize cultivars released between 1930 and 2010. The rates of P and K fertilizer application were those locally recommended for maize production in the area (P_2_O_5_ 85 _kg_ ha^−1^, K_2_O 90 kg ha^−1^). P (calcium superphosphate, 12% P_2_O_5_), K (KCl, 60% K_2_O), and N (urea, 46% N) fertilizers were applied to the ground before sowing. Each cultivar was planted in a replicated randomized block design with three replicates in 2010 and four replicates in 2011. The block size was 12 m^2^ with 5 rows × 0.6 m inter-row spacing × 4 m long.

**Table 1:** Characteristics of cultivars used in the experiments.

Cultivar Name	Year of release	Parents	Cultivar Type	Breeding institution	Period of duration (days)	Plant height (cm)
Jinhuanghou	1930	—	OPV	—	109	289
Xiaolihong	1940	—	OPV	—	107	217
Yinglizi	1943	—	OPV	—	109	277
Weier 156	1963	(WF9 × Os420) × (M14 × CI187-2)	Double crosses	—	106	278
Sishuang 1	1965	(Ying64 × Tie84) × (M14 × W20)	Double crosses	AAS in Siping, Jilin Province	106	258
Jidan 101	1967	Ji63 × M14	Single crosses	AAS in Jilin Province	111	271
Zhongdan 2	1973	Mo17 × Zi330	Single crosses	CAAS	121	289
Danyu 13	1979	Mo17 × E28	Single crosses	AAS in Dandong, Liaoning Province	114	269
Shendan 7	1982	5003 × E28	Single crosses	AAS in Shenyang, Liaoning Province	120	252
Yedan 4	1982	U8112 × Huangzao4	Single crosses	AAS in Laizhou, Shandong Province	105	281
Benyu 9	1982	7884Ht × MO17Ht	Single crosses	AAS in Benxi, Liaoning Province	102	259
Yedan 2	1983	Ye107 × Huangzao4	Single crosses	AAS in Laizhou, Shandong Province	111	271
Nongda 60	1985	5003 × Zong31	Single crosses	CAU	114	269
Yedan 13 *	1989	Ye478 × Dan340	Single crosses	AAS in Laizhou, Shandong Province	121	276
Yudan 18	1989	478You × Zheng22	Single crosses	AAS in Henan Province	116	257
Ludan 50	1990	Luyuan92 × Qi319	Single crosses	AAS in Shandong Province	121	268
Nongda 108	1991	178 × HuangC	Single crosses	CAU	118	256
Nongda 3138	1991	Zong31 × P138	Single crosses	CAU	119	286
Jidan 159	1994	Ji846 × Dan340	Single crosses	AAS in Jilin Province	109	268
Denghai 9	1995	DH65232 × 8723	Single crosses	AAS in Laizhou, Shandong Province	120	271
Shendan 16	1995	K12 × Shen137	Single crosses	AAS in Shenyang, Liaoning Province	119	296
Zhengdan 958 *	1996	Zheng58 × Chang7-2	Single crosses	AAS in Henan Province	118	252
Xianyu 335	2000	PH6WC × PH4CV	Single crosses	Pioneer Co., Ltd.	108	301
NE9	2007	T63 × Shen137	Single crosses	CAU	124	275

All cultivars used at Shun-Yi (SY), *cultivars used at Fu-Jia-Jie (FJJ). OPV refers to open-pollinated varieties, AAS refers to Academy of Agricultural Sciences, CAU to China Agricultural University; CAAS represents Chinese Academy of Agricultural Sciences and AAFS to Academy of Agricultural and Forestry Sciences.

Two cultivars, “Zhengdan 958” (ZD958) and “Yedan 13” (YD13), representing most widely cultivated new- and old-era cultivars, respectively, were planted in Fu-Jia-Jie, near Siping (43°17’N, 124°26’E). Five rates (0, 60, 120, 180, and 240 kg N ha^−1^) of N fertilizer application took place at Fu-Jia-Jie to study the effects of N application rate on grain nutrient concentrations between old- and new-ear cultivars. The rates of P and K fertilizer application were those locally recommended for maize production in the area (P_2_O_5_ 76 _kg_ ha^−1^, K_2_O 100 kg ha^−1^). P and K fertilizers, as described above, were applied to the prepared site before sowing, while the N fertilizer (as above) was applied in two splits (1:1), one before sowing and the other at the V8 stage. Each cultivar was planted using a split-plot experimental design. N fertilizer treatments took place in the main plots and the cultivars were grown in the subplots with four replicates during both 2010 and 2011. The block size was 15 m^2^ with 5 rows × 0.6 m inter-row spacing × 5 m long.

Plants were grown at a density of 60,000 plants ha^−1^. The dates of maize sowing and harvest were shown in **Table 2**. Maize was sown by hand and rain-fed during the growing season. Before sowing in 2010, six soil samples were taken from the uppermost soil layer (0 to 30 cm) at each location, mixed, used to measure the soil properties. The soil types in Shun-Yi and Fu-Jia-Jie were calcareous alluvial soil and sandy soil, respectively. After air-drying and removing debris and plant material, each sample was sieved through a 2-mm mesh for chemical analysis (**Table 2**). Organic matter was determined using the K_2_Cr_2_O_7_ method (Walkley, 1947) and total nitrogen (N) using the Kjeldahl procedure (Bremner, 1996). Available P (Olsen-P) was extracted with NaHCO_3_ and determined by spectrophotometry (Olsen et al., 1954). Available K was extracted with NH_4_OAc and determined by flame photometry (Van Reeuwijk, 1992). Soil pH was determined with a pH meter (water: soil was 2.5∶1) and mineralized N (Nmin, nitrate + ammonium) was extracted with 0.01 mol L^−1^ CaCl_2_ and determined with a flow analyzer (**Table 2**).

**Table 2:** Properties of soils in the field used for the experiments at Shun-Yi (SY) and Fu-Jia-Jie (FJJ).

Location	Soil type	Organic matter (g kg^−1^)	Total N (g kg^−1^)	Olsen-P (mg kg^−1^)	Available K (mg kg^−1^)	pH (1:2.5 w v^−1^)	Nmin (mg kg^−1^)	Sowing	Harvest
Shun-Yi (SY)	Calcareous alluvial soil	16.75	1.07	41.33	129	7.44	32.14	2010/5/20	2010/9/23
								2011/5/4	2011/9/10
Fu-Jia-Jie (FJJ)	Sandy soil	8.30	0.66	30.97	100	6.24	59.16	2010/5/7	2010/9/25
								2011/5/8	2011/9/27

### Plant Sample Harvest and Nutrient Analysis

At harvest, five ears were collected at random from the central area of each block. Grain yield was expressed at a 14% moisture content. Twenty kernels were collected from the middle portion of each ear, washed with deionized water and oven-dried at 70°C to determine kernel dry weight. The hundred-grain weight was derived by converting dry weight to 14% moisture content. The kernels were ground with a stainless-steel grinder GENO-2000 (Spex, Pittsburg, PA, USA); an 0.5 g aliquot of kernel powder was weighed into a glass tube and digested with HNO_3_-H_2_O_2_ in a microwave-accelerated reaction system (CEM; Matthews, NC, USA), according to the manufacturer’s instructions. The concentrations of Fe, Mn, Cu, Zn, Mg, K, and P in the digested solutions were determined by inductively coupled plasma atomic emission spectroscopy (ICP-OES; Perkin-Elmer, USA). Another 0.5 g powder aliquot was digested with concentrated sulfuric acid to determine the N concentration using the Kjeldahl method (Nelson and Somers, 1973).

### Data Analysis

The data collected from all locations, including genotypes, years, N rates (if applicable), and replicates were used for the interaction analysis of variance using the SAS statistics system (SAS Institute, Cary, NC, USA). These data meet the normal distribution and homogeneity of variance using Levene’s test. Two-way analysis of variance (ANOVA) was used to evaluate significant differences across genotypes and years (**Table S1**), and three-way analysis of variance for significant differences across genotypes, years, and N rates (**Table 5**). Years and N levels were treated as fixed, and genotypes were treated as random. Differences among genotypes were compared using the Tukey test at the significance threshold of *P* ≤ 0.05. Simple main effects analysis and Pearson correlation coefficients were calculated using SPSS Statistics 17.0 (SPSS, Inc., Chicago, IL, USA).

## Results

### Changes in Grain Yields and Nutrient Concentrations of Maize Cultivars Released Between 1930 and 2010 Under Sufficient N Rate

Twenty-four maize cultivars released from 1930 to 2010 in China were grown at Shun-Yi (SY) across 2 years (2010 and 2011) to examine the effects of breeding on grain yield and the concentration of each nutrient. N was applied at a rate of 240 kg ha^−1^. The ANOVA results indicated significant effects of years, genotypes on maize grain yield, hundred-grain weight, and eight nutrient concentrations of grain (**Table S1**). These results implied that grain nutrient concentrations were significantly affected by changes in genotypes and environments (mainly climatic changes from different years).

**Figure 1:**
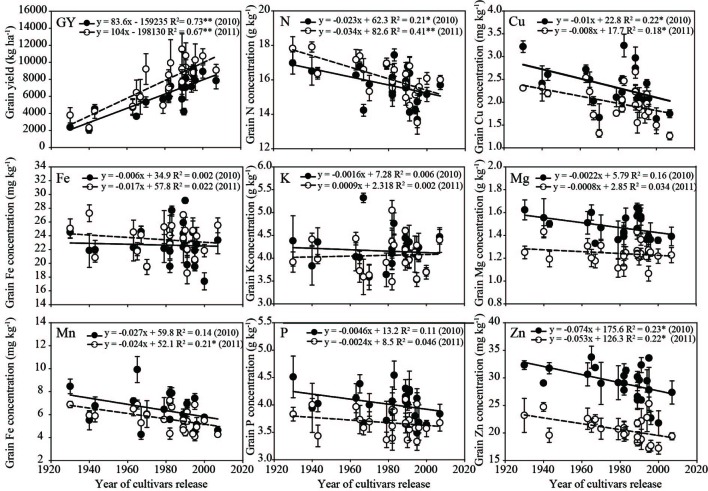
Relationship between the year of cultivars release, grain yield, and grain nutrient (N, Cu, Fe, K, Mg, Mn, P, and Zn) concentrations. Data were collected in Shun-Yi (SY). Closed and open circle denote the mean ± SD of three and four replicates in 2010 and 2011, respectively. *, ** denote significance at the 0.05 and 0.01 probability levels, respectively; NS, not significant (P > 0.05).

To understand the relationship between the years, grain yields and nutrient concentrations in relation to the cultivars released in different decades, the 2-year plant growth data were illustrated as shown in . Starting from the initial cultivar released in the 1930s, Jinhuanghou, the grain yield increased rapidly with increasing year of cultivar release, i.e. the newer the cultivar the higher the yield, showing a linear improvement of 83.6 kg ha−1 year−1 in 2010 and 104 kg ha−1 year−1 in 2011. There was a negative correlation between N, Cu, Mn, and Zn concentrations in the grain and the year of cultivar release. The decreases in concentrations of these four nutrients in the grain were 0.0285, 0.009, 0.0255, and 0.0635 mg kg−1 year−1, respectively. Although the Fe, K, Mg,and P concentrations in the grain decreased slightly over time of release, the differences in concentrations between release years were not significant (**Figure 1**).

The average grain yield estimated from the sampled plots ranged between 2059 and 9210 kg ha^−1^ in 2010 and between 2312 and 11532 kg ha^−1^ in 2011 (**Table 3**). After 80 years of selection for high-yield cultivars maize in China, the nutrient concentrations in the grain were 13.69–17.44 g kg^−1^ in 2010 and 13.52–17.91 g kg^−1^ in 2011 for N; 1.64–3.24 mg kg^−1^ in 2010 and 1.26–2.66 mg kg^−1^ in 2011 for Cu; 4.27–9.93 mg kg^−1^ in 2010 and 4.09–7.24 mg kg^−1^ in 2011 for Mn; 21.81–33.75 mg kg^−1^ in 2010 and 17.19–25.31 mg kg^−1^ in 2011 for Zn. Grain yield, Cu and Mn concentrations exhibited the highest coefficient of variation values (CV%; 30.81–31.30, 18.55–18.81, and 17. 75–22.24%, respectively; **Table 3**).

**Table 3:** Statistics analysis of grain yield (GY), hundred grains weight (HGW), grain number per panicle (GN) and eight nutrients (N, Cu, Fe, K, Mg, Mn, P and Zn) concentration in maize grain at Shun-Yi (SY) across 2 years (2010 and 2011).

Year	GY (kg ha^−1^)		HGW(g)		N (g kg^−1^)		Cu (mg kg^−1^)		Fe (mg kg^−1^)		K (g kg^−1^)		Mg (g kg^−1^)		Mn (mg kg^−1^)		P (g kg^−1^)		Zn (mg kg^−1^)	
	2010	2011	2010	2011	2010	2011	2010	2011	2010	2011	2010	2011	2010	2011	2010	2011	2010	2011	2010	2011
Mean	6244 ± 1924	7829 ± 2451	31.39 ± 2.98	26.52 ± 3.66	15.73 ± 1.01	16.07 ± 1.03	2.31 ± 0.44	1.98 ± 0.37	22.65 ± 2.65	23.42 ± 2.29	4.10 ± 0.39	4.06 ± 0.37	1.47 ± 0.11	1.24 ± 0.09	6.36 ± 1.42	5.64 ± 1.00	4.01 ± 0.28	3.68 ± 0.22	29.14 ± 3.05	20.63 ± 2.22
Range	2059–9210	2312–11532	26.42–37.50	20.65–33.43	13.69–17.44	13.52–17.91	1.64–3.24	1.26–2.66	17.39–29.12	18.58–27.27	3.53–4.76	3.48–5.05	1.25–1.64	1.07–1.43	4.27–9.93	4.09–7.24	3.49–4.54	3.33–4.10	21.81–33.75	17.19–25.31
CV (%)	30.81	31.30	9.49	13.82	6.42	6.39	18.81	18.55	11.68	9.79	9.42	9.21	7.42	6.96	22.24	17.75	6.90	6.05	10.46	10.75
R^2^	0.97**		0.51**		0.57**		0.70**		0.28**		0.26*		0.37**		0.72**		0.28**		0.54**	

Mean shown are means for all tested cultivars ± SD; CV, coefficient of variation; correlation coefficients (R^2^) between each 2 years for GY, HGW and grain mineral concentration; * significant at the 0.05 probability level; ** significant at the 0.01 probability level.

Significant negative correlations were found between grain yield and several grain nutrient concentrations (**Table 4**). Levels of N and Zn were highly negatively correlated with grain yield in both trial years. Levels of Mg was highly negatively correlated with grain yield in 2010 (r = −0.58), but not in 2011 (r = 0.09). By contrast, all the grain nutrient concentrations were not significantly correlated with the hundred-grain weight. Only N concentration in the grains was significantly and negatively correlated with grain number across years (r = −0.53 to −0.72). Among the nutrients, N concentration was positively associated with Cu, Fe, Mg, Mn, P, and Zn concentrations in the grain (r = 0.43–0.64). For Mg, Mn, P, and Zn concentration, significant and positive correlations were observed between each other across 2 years (r = 0.42–0.85).

**Table 4:** Correlation coefficients (r) between grain yield (GY), hundred grains weight (HGW), grain number per panicle (GN), and mineral nutrients (N, Cu, Fe, K, Mg, Mn, P, and Zn) concentration in maize at Shun-Yi (SY) across 2 years (2010 and 2011).

	GY	HGW	GN	N	Cu	Fe	K	Mg	Mn	P	Zn
GY		0.68**	0.70**	−0.60**	−0.39	−0.25	−0.07	0.09	−0.31	−0.05	−0.46*
HGW	0.54**		0.12	−0.22	−0.31	−0.31	−0.28	0.19	0.06	0.09	−0.13
GN	0.84**	0.19		−0.72**	−0.31	−0.12	0.13	−0.23	−0.41*	−0.13	−0.34
N	−0.60**	−0.32	−0.53**		0.28	0.51*	0.07	0.43*	0.50*	0.44*	0.49*
Cu	−0.34	−0.27	−0.21	0.49*		0.13	0.11	0.11	0.36	0.30	0.20
Fe	−0.30	−0.02	−0.18	0.46*	0.45*		0.49*	0.29	0.04	0.34	0.04
K	−0.04	−0.19	0.15	−0.02	0.20	0.51*		0.41*	−0.11	0.56**	0.27
Mg	−0.58**	−0.17	−0.51*	0.64**	0.43*	0.38	0.19		0.28	0.79**	0.46*
Mn	−0.28	−0.27	−0.19	0.55**	0.46*	−0.13	−0.07	0.50*		0.54**	0.42*
P	−0.35	−0.15	−0.29	0.56**	0.60**	0.30	0.27	0.85**	0.71**		0.54**
Zn	−0.46*	−0.16	−0.4	0.35	0.34	0.26	0.26	0.36	0.49*	0.51*	

Data of 2010 are located at the left below diagonal, and those of 2011 at the right above diagonal; * significant at the 0.05 probability level; ** significant at the 0.01 probability level.

### Effects of Nitrogen Application Rate on Grain Nutrient Concentrations in the Most Widely Cultivated New- and Old-Era Maize Cultivars

Two cultivars, “Zhengdan 958” (ZD958) released in 1996, and “Yedan 13” (YD13) released in 1989, were the dominant Chinese cultivars used in their corresponding decades (**Table 1**; Chen et al., 2014). The YD13 and ZD958 presented as old-era and new-era cultivar, respectively (Ciampitti and Vyn, 2012). To investigate the responses of grain nutrient accumulation in different-ear cultivars to changes of N fertilizer inputs, YD13 and ZD958 were grown at Fu-Jia-Jie (FJJ) across 2 years (2010 and 2011) with supply of five different N rates (0, 60, 120, 180, and 240 kg N ha^−1^). The ANOVA results revealed significant effects of years, genotypes, and N rates on most of investigated traits (**Table 5**). Grain yield was not significantly affected by N×G interaction, suggesting both cultivars had similar yield responsiveness to changes of N rates. By contrast, the traits of GNC, GCuC, GFeC, GMgC, and GZnC showed significant N×G interactions. In addition, the GMnC did not significantly differ between two genotypes, and GKC and GKP did not significantly change across different N rates.

**Table 5:** Variance analysis (ANOVA) of the effects of year (Y), nitrogen (N) and genotypes (G) on maize grain yield and grain mineral nutrients (N, Cu, Fe, K, Mg, Mn, P and Zn) concentration (FJJ).

Treatments	GY (kg ha^−1^)	N (g kg^−1^)	Cu (mg kg^−1^)	Fe (mg kg^−1^)	K (g kg^−1^)	Mg (g kg^−1^)	Mn (mg kg^−1^)	P (g kg^−1^)	Zn (mg kg^−1^)
Genotype (G)									
ZD958	9147 ± 1609a	11.63 ± 1.76b	0.78 ± 0.16b	16.32 ± 1.87b	3.48 ± 0.21b	0.97 ± 0.11b	3.99 ± 0.59a	2.92 ± 0.39b	11.68 ± 2.02b
YD13	7295 ± 1426b	12.39 ± 2.03a	1.10 ± 0.16a	18.55 ± 3.05a	3.84 ± 0.15a	1.10 ± 0.09a	4.07 ± 0.48a	3.34 ± 0.36a	14.23 ± 1.86a
Nitrogen (N)									
N0	6135 ± 1715d	9.49 ± 1.51d	0.91 ± 0.18b	14.69 ± 1.00c	3.71 ± 0.19a	1.03 ± 0.07b	3.40 ± 0.39d	3.15 ± 0.29ab	16.10 ± 1.45a
N60	7744 ± 1401c	11.12 ± 1.34c	0.95 ± 0.20b	16.29 ± 1.49b	3.70 ± 0.23a	1.00 ± 0.11b	3.83 ± 0.31c	3.18 ± 0.38a	13.24 ± 1.78b
N120	8776 ± 1166b	12.55 ± 1.16b	1.04 ± 0.22a	18.40 ± 2.01a	3.63 ± 0.28a	1.02 ± 0.12b	4.29 ± 0.37ab	3.12 ± 0.41ab	11.73 ± 1.54c
N180	8961 ± 1246ab	13.38 ± 0.74a	0.91 ± 0.20b	18.98 ± 3.20a	3.66 ± 0.27a	1.07 ± 0.10a	4.22 ± 0.27b	3.14 ± 0.40ab	12.09 ± 1.59c
N240	9442 ± 1214a	13.47 ± 1.13a	0.89 ± 0.29b	18.82 ± 2.73a	3.60 ± 0.34a	1.08 ± 0.17a	4.41 ± 0.56a	3.06 ± 0.62b	11.62 ± 1.97c
Year (Y)									
2010	8709 ± 1251a	12.82 ± 1.69a	1.01 ± 0.15a	16.55 ± 1.95b	3.65 ± 0.23a	1.11 ± 0.09a	4.25 ± 0.47a	3.45 ± 0.24a	12.74 ± 2.00b
2011	7659 ± 1922b	11.22 ± 1.83b	0.87 ± 0.26b	18.32 ± 3.16a	3.66 ± 0.29a	0.97 ± 0.10b	3.81 ± 0.51b	2.80 ± 0.30b	13.17 ± 2.65a
Source of variation									
Y	**	**	**	**	ns	**	**	**	**
G	**	**	**	**	**	**	ns	**	**
N	**	**	**	**	ns	**	**	ns	**
N×G	ns	**	*	**	ns	*	ns	**	*

Data shown are means for all tested cultivars ± SD; the numbers followed by different letters indicate significant differences (P < 0.05). *Significant at the 0.05 probability level; ** significant at the 0.01 probability level; ns, not significant (P > 0.05).

Grain yield of ZD958 was 25.39% higher than that of YD13, while ZD958 had lower grain nutrient concentrations than did YD13, except for Mn, with reduction of GNC, GCuC, GFeC, GKC, GMgC, GMnC, GPC, and GZnC at 6.13%, 29.09%, 12.02%, 9.38%, 11.82%, 1.97%, 12.57%, and 17.92%, respectively (**Table 5**). Grain yield, GNC, GFeC, GMgC, and GMnC increased with N application rates while GZnC decreased. The CV% values for all minerals were shown in **Table S2**. Simple main effects analysis showed the grain Cu and Zn concentrations were lower in the new- than in the old-era cultivars, regardless of N application rates. Changes in grain N, Fe, Mg, and P concentrations appeared to be not significant at N0 (**Table S3**).

**Figure 2:**
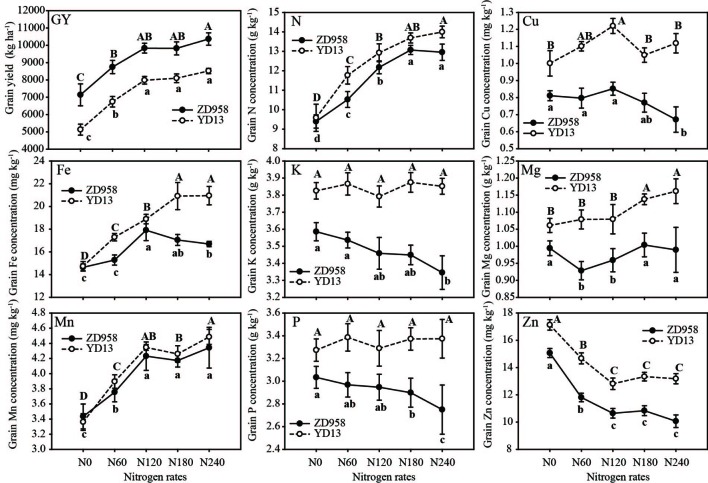
Grain yield and grain nutrient (N, Cu, Fe, K, Mg, Mn, P, and Zn) concentrations in response to nitrogen treatment in hybrids ZD958 (closed circle) and YD13 (open circle). Data were collected in Fu-Jia-Jie (FJJ). N0, N60, N120, N180, and N240 represent N rates at 0, 60, 120, 180, and 240 kg ha^−1^, respectively. Data shown are means for cultivar in 2010 and 2011 ± SD (n = 8). Upper- and lowercase letters denote the results of t-test to YD13 and ZD958 at five N rates, respectively. Any two samples with a common letter are not significantly different (P > 0.05).

With the increases of N inputs, grain yields of ZD958 and YD13 were increased with the same trends (**Figure 2**). The yields had reached the maximum levels at N120 which is indicative of the optimal N inputs for yield formation. By contrast, GNC reached the maximum levels in at N180. Grain accumulation for some nutrients showed different responsive pattern between these two cultivars. In old-era cultivar YD13, GFeC continuously increased to the pick level at N180. In new-ear cultivar ZD958, the increases of GFeC by N rates were saturated at N120 and GFeC further declined under high N input (N240). The similar patterns were also observed for GKC, GPC, and GCuC. Moreover, the declines of GZnC were observed with the increase. However, GZnC showed an opposite response, with the highest value at N0 declining to a stable value at N120 in both cultivars. Thus, for new-era cultivar ZD958, the optimal N rate (N120) was essential to achieve the highest grain nutrient concentration, particularly for GFeC, without decrease of grain yield.

## Discussion

Grain yield increased rapidly with the year of cultivars release at a linear improvement of 93.8 kg ha^−1^ year^−1^ (**Figure 1**). This result is in agreement with other reports stating that maize yield has risen steadily in China, America, France, Canada, and Argentina since the Green Revolution (Ci et al., 2011; Ciampitti and Vyn, 2012), mainly due to improved genetics and management practices (Duvick, 2005). In this work, the concentrations of grain N, Cu, Mn, and Zn have significantly decreased since the first maize cultivars were released in China in the 1930s. The Fe, K, Mg, and P concentrations, however, were not significantly affected by the year of the cultivars’s release, in spite of a trend for decreasing concentration over time. Over the past 50 years, the protein, Fe, and Zn content of cereals (maize, rice, wheat, barley, oats, millet, rye and sorghum) has declined as production has increased (DeFries et al., 2015). Similarly, grain concentrations of N, P, Mg, Mn, and Zn have decreased with rises in plant density between 1960 and 1980 in the USA (Vyn and Tollenaar, 1998). Significant causative factors for the downward trend of nutrient concentrations in wheat grain are increasing yield and harvest index (Fan et al., 2008).

Increased yield was the important index for maize breeding in Chinese cultivars and this resulted in changes to grain quality occurring at faster rates during recent decades (Li et al., 2016). In the present study, significant negative correlations were found between grain yield and several grain nutrients (Zn, Mg, and N) concentrations (**Table 4**). With improvements to yield between 1934 and 2013, traits such as kernel number and kernel mass per ear increased, whereas N concentration in grains decreased (DeBruin et al., 2017). We found all the grain nutrient concentrations were not significantly correlated with hundred-grain weight. N concentrations in the grain were significantly and negatively correlated to grain number. Other studies had found that planting density and planting density × environment with grain protein concentration exhibited more significant interactions in Chinese cultivars (Li et al., 2016). Variance analysis showed that the grain nutrient concentrations were significantly affected by genetics, the environment (years, N levels), and their interactions. Similar results have been reported by several other research groups (Simic et al., 2009; Prasanna et al., 2011).

This study revealed significant positive correlations between nutrient concentrations in maize grains (**Table 4**), as had been reported in wheat and rice grains (Skrbic and Onjia, 2007; Cakmak et al., 2010; Chatzav et al., 2010; Anandan et al., 2011). One study reported that Cu, Fe, Mn, and Zn concentrations in grains of 297 genotypes of maize measured over 2 years showed positive associations between individual micronutrient concentrations (Simic et al., 2009). The underlying quantitative trait loci of kernel weight, nutrient concentrations (Mg, Mn) and contents (Zn, K, Mg, P) were identified in a maize recombinant inbred population. These traits were significantly affected by genotype, location, and year (Gu et al., 2015). We found that N concentrations in the grain were significantly and positively correlated with Fe, Mn, Mg, and P concentrations, while Cu and Zn levels were positively correlated with N concentration in only 1 year of the experiment. Using quantitative trait loci in wheat, a previous study identified a relationship between grain protein concentration and grain Fe, Cu, and Zn concentrations (Peleg et al., 2009). Additionally, it was discovered that an Fe-regulated transporter (IRT1) plays an important role in Mn loading of grains in barley (Long et al., 2018), suggesting that there may be a common physiological mechanism for the accumulation of nutrients in grains. Thus, the concentrations of several nutrients can be increased as a whole without affecting crop yield.

Results from the present study showed that the new-era cultivars had higher grain yields and lower grain nutrient concentrations (N, Fe, Zn, Mg, and Cu) than did the old-era cultivars (**Table 5**). Consistent with our results, a review by Ciampitti and Vyn (2012) reported greater grain yield and N-use efficiency for varieties bred in 1940–1990 compared to varieties bred in 1991–2011. Grain N concentrations, however, decreased with the year of release (Duvick, 2005; Chen et al., 2013; Haegele et al., 2013). In our study, the new-era cultivars exhibited higher grain yields and lower GNC, GCuC, GFeC, GMgC, and GZnC than the old-era varieties. The grain yield of new variety ZD958 was 25.39% higher than that of old variety YD13, while GNC, GCuC, GFeC, GMgC, and GZnC of ZD958 were all lower. Compared with 1961, the protein, Fe and Zn concentrations of eight cereals, including maize, in 2011 were 4%, 19%, and 5% lower, respectively (DeFries et al., 2015). Differences in genotype can lead to changes in micronutrient concentrations and bioavailability (Oikeh et al., 2003a).

The dilution effect of essential nutrients such as N, Fe, and Zn is due to the large increases in yield of most recent genotypes (Fan et al., 2008; Cakmak and Kutman, 2018). Environmental and genetic factors impacted dilution effect (Davis, 2005). High-yield high-Zn cultivars were chosen from 123 modern wheat cultivars by Wang et al. (2018). The effect of yield dilution is not necessarily an obstacle for increasing Zn concentration in grain (Wang et al., 2018). Breeding efforts that select for increased GN and GW should increase yield (Haegele et al., 2013; DeBruin et al., 2017). The yield increase was most strongly correlated to GN and HGW, negative correlation with N and Zn concentration (**Table 4**). Nutrient content (N, P, and K) appears to be mostly dictated by dry matter production with development of new hybrids (Woli et al., 2017). **Figure S1** showed there was a positively correlation between N, Cu, Fe, K, Mg, P, and Zn contents in the grain with cultivars development. Grain nutrient content increased with GW, but their growth rates were lower than that of grain weight. This may be attributed to breeders selecting exclusively for grain yield, but not grain quality, which has contributed to the reduction in micronutrient concentrations even in recent years (Fan et al., 2008; Gu et al., 2015; **Figure 1**). Crops that were directly selected for reduced N concentration may have improved yield due to the greater energy requirement to produce proteins compared with carbohydrates (DeBruin et al., 2017). The decrease in grain quality coincided with the introduction and extensive use of tropical germplasm into China from American maize during the 1970s and 1980s (Li et al., 2016). Post-silking uptake and remobilization of some nutrients (e.g. N, P and Zn) from vegetative tissues can result in high harvest index values (Bender et al., 2013). A previous study had suggested that GNC is affected by genotype × environment, which is mainly reflected in N remobilization (Chen et al., 2014). The decreases in Fe, Mn, Cu, and Zn concentrations in maize grains intercropped with legumes is caused mainly by the decrease in transfer of nutrients from vegetative organs to grains (Xia et al., 2013). In very recent years, new varieties bred with increased stress tolerance are more likely to achieve high yields under low or high N conditions (Chen et al., 2013). Nevertheless, modern maize cultivars with higher yields tend to result in decreased grain mineral concentrations (Feil et al., 2005). This suggested that in modern maize cultivars decline of N, Fe, and Zn concentration may result from breeding effects, while decrease of Cu and Mn from dilution effects by improved grain biomass. Therefore, maize breeders should pay more attention to selecting grain N, Fe, and Zn accumulation, allowing breed ideal cultivars with high yield and high quality simultaneously.

High N rates characterize modern crop production and influence the accumulation of other nutrients (Hamnér et al., 2017). As found in this study and others, the P, K, Mg, Fe, Zn, Mn, and Cu concentrations of grain are primarily influenced by the N application rate or N × environment at maturity (Ciampitti et al., 2013; Ciampitti and Vyn, 2013; **Table 5**). In the present study, positive correlations were observed between GNC and GFeC, GMgC, GMnC, or GPC. It was shown that Cu, Fe, and Mn concentrations rose as N application rate increased, while K, P, Zn, and Mg concentrations decreased. Our results indicate that N fertilization has a synergistic effect on grain nutrient concentrations. Similarly, the decrease in P and K concentrations with increased N fertilization in maize has previously been reported (Riedell, 2010). High N concentrations had a positive effect on Fe, Zn, Cu, and Mn concentrations in wheat grain (Hamnér et al., 2017). The distinct soil Zn availabilities affect Zn accumulation in maize grain strongly (Gu et al., 2015). In recent decades, although concentrations of Zn, Fe, Cu, and Mg in soil have either increased or remained stable, the micronutrient concentrations in wheat grain have decreased (Fan et al., 2008). Further, application of urea usually causes soil acidification, helps to accelerate nutrient leaching and changes nutrient forms/availability, resulting in reduced nutrient accumulation in grain as N application rates increase (Guo et al., 2010).

Our results demonstrate that high N application rates induce the decrease in GCuC, GFeC, GKC, and GPC in modern varieties, but not in the old varieties, suggesting that new varieties respond more to increased N levels (**Figure 2**). The dilution effect of Zn in maize grains is caused by higher yield resulting from N fertilization (Feil et al., 2005). GNC and GMnC rose with an increase in N rates in both old and new cultivars. Maize nutrient (P, K, S, Ca, Mg, Fe, and Zn) harvest indices, nutrient contents, and internal use efficiencies at maturity responded predominantly to N application rates (Ciampitti and Vyn, 2013; Ciampitti et al., 2013). Recent studies have shown that N fertilization can significantly increase Fe and Zn contents in wheat grains under greenhouse and field conditions (Cakmak et al., 2010; Kutman et al., 2010; Shi et al., 2010). We found that GFeC rose with an increase in N application rates, but decreased with excessive fertilization, particularly in new cultivars. Moreover, a highly negative correlation between N rates and mineral concentrations in maize grains was reported by Rui et al. (2009). These results suggest that the discrepancies observed in different studies are likely to be associated with variations in the uptake and internal use efficiency of N among different genotypes and experimental conditions (Ma and Zheng, 2018). The mechanism by which GCuC, GPC, GKC, and even GFeC decreased with high N rates in new cultivars requires further study.

Optimized N application can increase maize yield, kernel weight, and grain concentrations of Zn, Fe, Mn, and Cu (Haegele et al., 2013; Xue et al., 2014). Thus, the selection of high-yielding maize varieties in combination with precise N management can increase grain N concentration without negative effects on yield (Chen et al., 2015). Crop nutrition management accommodates not only the demand for N, but also for other nutrients (Hamnér et al., 2017). Our results indicate that a suitable N rate (120–180 kg N ha^−1^) can increase N, Fe, Mg, and Mn concentrations in modern varieties without harming grain yield. Increased N application rates reduced Cu, Zn, P, and K concentrations in the modern varieties. Therefore, an optimized N management strategy is needed to enrich grains with essential mineral nutrients while still achieving higher grain yield in modern maize cultivars.

## Data Availability Statement

All datasets generated for this study are included in the article/**Supplementary Material**.

## Author Contributions 

LYu and RG initiated and designed the experiments. SG, YanhC, XC, YanlC and LYa performed the field experiments. LW and YQ helped in statistical analysis. ML and FC provided the material. SG wrote the manuscript. GM, RG and LYu revised the manuscript.

## Conflict of Interest

The authors declare that the research was conducted in the absence of any commercial or financial relationships that could be construed as a potential conflict of interest.

## Supplementary Material

The Supplementary Material for this article can be found online at: https://www.frontiersin.org/articles/10.3389/fpls.2019.01662/full#supplementary-material

Figure S1 Relationship between the year of cultivars release, grain yield, and grain nutrient (N, Cu, Fe, K, Mg, Mn, P and Zn) concentrations. Data were collected in Shun-Yi (SY). Closed and open circle denote the mean ± SD of three and four replicates in 2010 and 2011, respectively. *, ** denote significance at the 0.05 and 0.01 probability levels, respectively.
