# Supplementary figures and images for: Grain Mineral Accumulation Changes in Chinese Maize Cultivars Released in Different Decades and the Responses to Nitrogen Fertilizer
Source: Front Plant Sci. 2020 Jan 14;10:1662. doi: 10.3389/fpls.2019.01662 (PMC6971105; doi:10.3389/fpls.2019.01662)

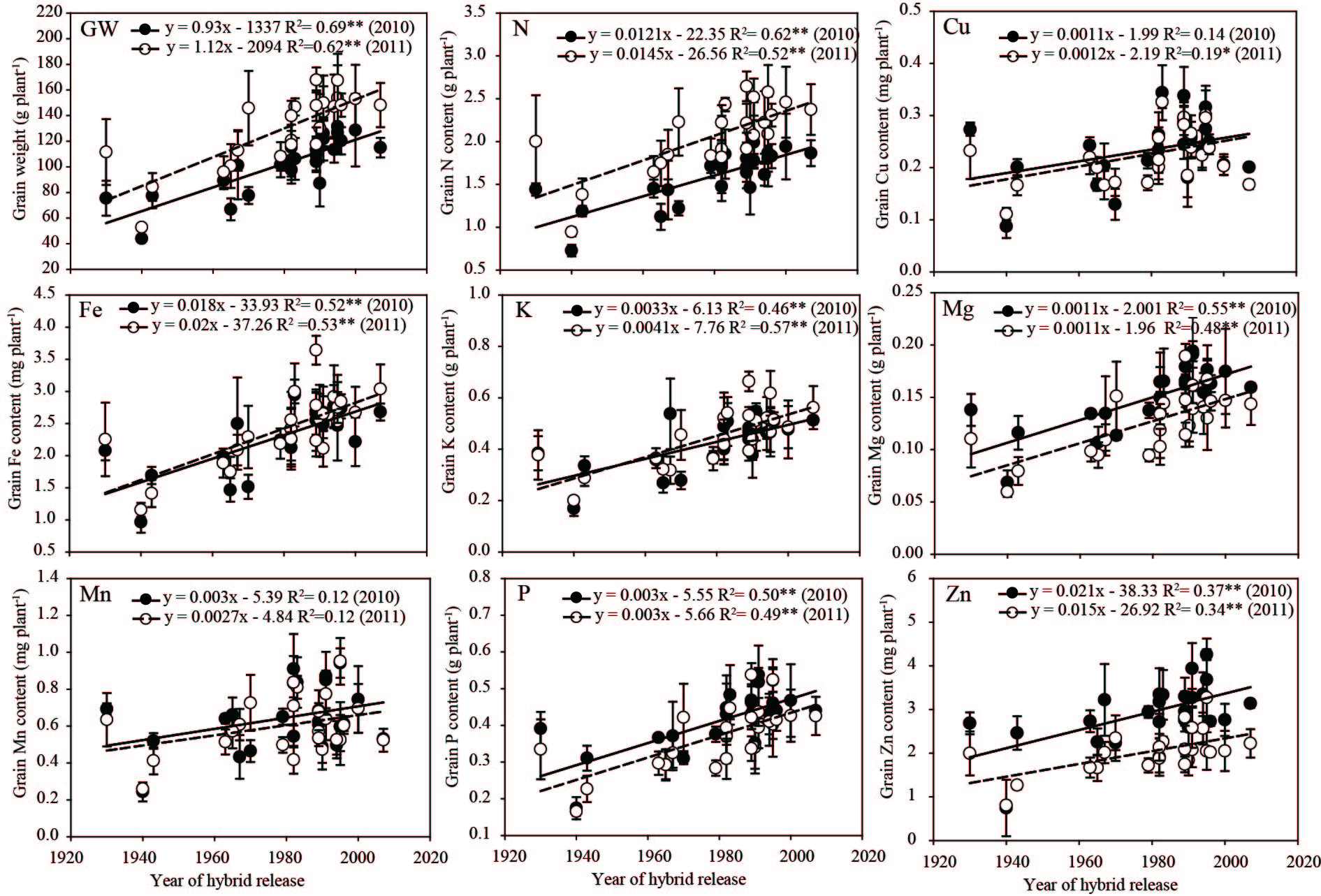

Supplement: Figure S1 — Relationship between the year of cultivars release, grain yield, and grain nutrient (N, Cu, Fe, K, Mg, Mn, P and Zn) concentrations. Data were collected in Shun-Yi (SY). Closed and open circle denote the mean ± SD of three and four replicates in 2010 and 2011, respectively. *, ** denote significance at the 0.05 and 0.01 probability levels, respectively. [file Image_1.jpeg]
