# Supplementary material for: Grain Mineral Accumulation Changes in Chinese Maize Cultivars Released in Different Decades and the Responses to Nitrogen Fertilizer
Source: Front Plant Sci. 2020 Jan 14;10:1662. doi: 10.3389/fpls.2019.01662 (PMC6971105; doi:10.3389/fpls.2019.01662)
Supplement: Supplementary file 2 [file Table_1.docx]

Table S1 Variance analysis (ANOVA) of the effects of years and genotypes on maize grain yield (GY), hundred grains weight (HGW), grain number (GN) and concentration of grain mineral nutrients (N, Cu, Fe, K, Mg, Mn, P and Zn) in Shun-Yi (SY) across two years (2010 and 2011).

| Source of variation | GY  (kg ha^-1^) | HGW  (g) | GN  (ear^-1^) | N  (g kg^-1^) | Cu  (mg kg^-1^) | Fe  (mg kg^-1^) | K  (g kg^-1^) | Mg  (g kg^-1^) | Mn  (mg kg^-1^) | P (g kg^-1^) | Zn  (mg kg^-1^) |
| --- | --- | --- | --- | --- | --- | --- | --- | --- | --- | --- | --- |
| Year | 100361130** | 964.46** | 1060673.69** | 13.39** | 10.89** | 612.30** | 17.79** | 9.85** | 118.53** | 53.60** | 7094.95** |
| Genotype | 27843386** | 64.45** | 28395.54** | 7.60** | 0.94** | 26.66** | 0.53** | 0.04** | 8.02** | 0.27** | 33.79** |

Note: Data shown are mean square; ** significant at the 0.01probability level.

Table S2 Descriptive statistics of eight nutrients (N, Cu, Fe, K, Mg, Mn, P and Zn) concentration in maize grain in Fu-Jia-Jie (FJJ).

|  |  | N0 | |  | N60 | |  | N120 | |  | N180 | |  | N240 | |
| --- | --- | --- | --- | --- | --- | --- | --- | --- | --- | --- | --- | --- | --- | --- | --- |
|  | Year | Mean | CV (%) |  | Mean | CV (%) |  | Mean | CV (%) |  | Mean | CV (%) |  | Mean | CV (%) |
| N (g kg^-1^) | 2010 | 10.46±1.57 | 14.98 |  | 12.23±0.85 | 6.92 |  | 13.48±0.86 | 6.35 |  | 13.85±0.61 | 4.43 |  | 13.95±1.31 | 9.42 |
|  | 2011 | 8.52±0.52 | 6.05 |  | 10.06±0.66 | 6.55 |  | 11.63±0.44 | 3.79 |  | 12.90±0.54 | 4.20 |  | 13.00±0.71 | 5.43 |
| Cu (mg kg^-1^) | 2010 | 1.02±0.16 | 15.99 |  | 1.02±0.16 | 15.6 |  | 1.03±0.17 | 16.49 |  | 0.99±0.15 | 14.68 |  | 0.99±0.16 | 16.39 |
|  | 2011 | 0.79±0.13 | 15.95 |  | 0.88±0.23 | 25.59 |  | 1.04±0.28 | 26.43 |  | 0.83±0.22 | 26.28 |  | 0.80±0.37 | 46.30 |
| Fe (mg kg^-1^) | 2010 | 14.33±0.93 | 6.52 |  | 15.80±1.72 | 10.87 |  | 17.36±1.51 | 8.67 |  | 17.00±1.66 | 9.77 |  | 18.25±1.32 | 7.26 |
|  | 2011 | 15.04±0.99 | 6.57 |  | 16.77±1.13 | 6.71 |  | 19.43±1.99 | 10.22 |  | 20.97±3.19 | 15.21 |  | 19.40±3.67 | 18.94 |
| K (g kg^-1^) | 2010 | 3.69±0.14 | 3.81 |  | 3.67±0.25 | 6.90 |  | 3.54±0.32 | 9.10 |  | 3.62±0.25 | 6.80 |  | 3.75±0.18 | 4.70 |
|  | 2011 | 3.72±0.23 | 6.25 |  | 3.74±0.22 | 5.78 |  | 3.71±0.21 | 5.57 |  | 3.70±0.30 | 8.23 |  | 3.45±0.40 | 11.59 |
| Mg (g kg^-1^) | 2010 | 1.05±0.05 | 4.66 |  | 1.07±0.09 | 8.05 |  | 1.11±0.09 | 7.76 |  | 1.13±0.06 | 5.76 |  | 1.20±0.07 | 5.41 |
|  | 2011 | 1.00±0.08 | 7.56 |  | 0.94±0.08 | 8.76 |  | 0.93±0.07 | 7.93 |  | 1.02±0.10 | 10.18 |  | 0.95±0.15 | 15.39 |
| Mn (mg kg^-1^) | 2010 | 3.70±0.28 | 7.47 |  | 4.03±0.27 | 6.78 |  | 4.54±0.22 | 4.92 |  | 4.19±0.27 | 6.48 |  | 4.77±0.40 | 8.31 |
|  | 2011 | 3.10±0.18 | 5.75 |  | 3.62±0.18 | 4.84 |  | 4.04±0.33 | 8.19 |  | 4.24±0.29 | 6.74 |  | 4.06±0.47 | 11.53 |
| P (g kg^-1^) | 2010 | 3.39±0.13 | 3.95 |  | 3.46±0.26 | 7.63 |  | 3.45±0.27 | 7.80 |  | 3.40±0.25 | 7.47 |  | 3.56±0.28 | 7.74 |
|  | 2011 | 2.91±0.19 | 6.46 |  | 2.89±0.23 | 7.83 |  | 2.79±0.20 | 7.33 |  | 2.87±0.33 | 11.36 |  | 2.57±0.44 | 17.02 |
| Zn (mg kg^-1^) | 2010 | 15.43±1.18 | 7.65 |  | 12.76±1.63 | 12.81 |  | 11.14±1.65 | 14.83 |  | 12.21±1.54 | 12.61 |  | 12.17±1.15 | 9.47 |
|  | 2011 | 16.76±1.45 | 8.64 |  | 13.72±1.89 | 13.78 |  | 12.31±1.26 | 10.22 |  | 11.96±1.73 | 14.46 |  | 11.07±2.51 | 22.70 |

Note: Mean shown are means for all tested cultivars ± SD (Standard Deviation); CV, coefficient of variation. Table S3 Simple main effects analysis of nitrogen and genotypes on maize grain mineral nutrients (N, Cu, Fe, Mg, P and Zn) concentration (FJJ).

| Treatments | N (g kg^-1^) | Cu (mg kg^-1^) | Fe (mg kg^-1^) | Mg (g kg^-1^) | P (g kg^-1^) | Zn (mg kg^-1^) |
| --- | --- | --- | --- | --- | --- | --- |
| N0 | -0.19±0.64 | -0.19±0.075* | -0.089±0.88 | -0.067±0.048 | -0.24±0.19 | -2.07±0.56** |
| N60 | 0.33±0.66 | -0.42±0.075** | -1.88±0.88* | -0.17±0.048** | -0.67±0.19** | -4.40±0.56** |
| N120 | -1.69±0.64* | -0.42±0.075** | -2.81±0.88** | -0.13±0.048* | -0.33±0.19 | -1.89±0.56** |
| N180 | -1.21±0.64* | -0.20±0.075* | -3.14±0.88** | -0.16±0.048** | -0.43±0.19* | -1.99±0.56** |
| N240 | -1.01±0.64 | -0.37±0.075** | -3.28±0.88** | -0.12±0.048* | -0.43±0.19* | -2.36±0.56** |

Note: Value is mean difference (ZD958-YD13) ± SE (Standard Error);* significant at the 0.05 probability level; ** significant at the 0.01probability level.
